# Mercury poisoning complicated by acquired neuromyotonia syndrome: A case report

**DOI:** 10.1097/MD.0000000000026910

**Published:** 2021-08-13

**Authors:** Enrong Ran, Maohe Wang, Yanxia Yi, Mei Feng, Yuanjun Liu

**Affiliations:** a Department of Nephrology; b Department of Hepatobiliary Surgery, Suining Central Hospital, Suining, Sichuan Province, China.

**Keywords:** acquired neuromyotonia syndrome, case report, heavy metal poisoning, hyponatremia, mercury poisoning

## Abstract

**Rationale:**

Acquired neuromyotonia syndrome is a rare form of peripheral nerve hyperexcitability syndrome. It is characterized by spontaneous and continuous muscle contractions. Acquired neuromyotonia syndrome is mainly observed in patients with autoimmune diseases or tumors, but it is a rare neurological clinical manifestation in patients with mercury poisoning.

**Patient concerns:**

A 56-year-old woman presented with continuous and involuntary muscle twitching in her legs for 2 months; it was accompanied by a burning sensation in the lower limbs, insomnia, fatigue, and night sweats. These symptoms did not disappear during sleep.

**Diagnoses:**

Toxicological blood analysis via atomic fluorescence spectrometry revealed that the level of mercury was 0.07 μmol/L (normal level: <0.05 μmol/L). Her urinary mercury level measured using the cold atomic absorption method was 217.50 μmol/mol creatinine, which was considerably higher than the reference range (0–2.25 μmol/mol creatinine for people not in contact with mercury, 0–20 μmol/mol creatinine following long-term exposure). Upon further testing, a high level of mercury (10,572 mg/kg) was detected in the patient's cream. Accordingly, this patient was diagnosed with mercury poisoning.

**Interventions:**

Treatment with 2,3-dimercapto-1-propanesulfonic acid (DMPS) was initiated. Her urinary mercury level decreased to 9.67 μmol/mol creatinine, and her neuromyotonia syndrome and hyponatremia were relieved, with urine protein completely disappearing after 3 months of treatment.

**Outcomes:**

After DMPS treatment, the clinical manifestations of the nervous system disappeared and electrolyte parameters returned to normal levels.

**Lessons:**

Acquired neuromyotonia syndrome is a rare disorder caused by the hyperexcitability of peripheral nerves, resulting in spontaneous and continuous muscle contraction. Mercury poisoning should be considered in patients with neuromyotonia syndrome. Early detection of mercury poisoning can prevent unnecessary examinations and treatments.

## 1 Introduction

Acquired neuromyotonia syndrome is a rare form of peripheral nerve hyperexcitability that was first described by Hyam Isaacs in the 1960s.^[1]^ It is characterized by spontaneous and continuous muscle contractions. Acquired neuromyotonia syndrome is mainly observed in patients with autoimmune diseases or tumors.^[2–4]^ Mercury exerts negative effects on the nervous, urinary, digestive, endocrine, and respiratory systems.^[5,6]^ Neurological symptoms of mercury poisoning include sensory disturbance, tremor, and cognitive impairment.^[7]^ However, neuromyotonia syndrome is rarely mentioned. Here, we describe a patient with occult mercury exposure who presented with acquired neuromyotonia syndrome and hyponatremia that was difficult to treat.

## 2 Case presentation

A 56-year-old woman presented with continuous and involuntary muscle twitching in her legs for 2 months, accompanied by a burning sensation in the lower limbs, insomnia, fatigue, and night sweats; these symptoms did not disappear even when she was asleep. She denied having fever, joint pain, muscle weakness, cough, abdominal pain, diarrhea, and vomiting. Physical examination showed persistent involuntary peristalsis in the bilateral calf muscles, observed as a continuous, undulating, and wave-like rippling of the muscles. Laboratory investigation was normal for electrolytes: potassium 3.63 mmol/L (normal range: 3.50–5.50 mmol/L), sodium 139.5 mmol/L (normal range: 135–145 mmol/L), chlorine 97 mmol/L (normal range: 96–108 mmol/L), calcium 2.6 mmol/L (normal range: 2.25–2.58 mmol/L), phosphorus 1.81 mmol/L (normal range: 0.96–1.80 mmol/L), and magnesium 1.01 mmol/L (normal range: 0.65–1.15 mmol/L). Her complete blood cell count, liver enzymes, renal function, blood glucose level, thyroid hormone levels, immunoglobulin, anticardiolipase A2 antibody, antinuclear antibody, rheumatoid factor, and complement C3/C4 levels were normal. Test results for hepatitis B and C viruses and human immunodeficiency virus were negative. Routine urine analysis showed the following results: pH 6, specific gravity 1.025, and 24-h urine protein 2.04 g. The kidney ultrasound results were normal. Brain magnetic resonance imaging, electroencephalogram, and peripheral nerve conduction velocity were normal. A fascicular fibrillation potential was observed in the relaxed state of the bilateral gastrocnemius muscles. On the basis of the symptoms and electromyography (EMG) records, the patient was diagnosed with acquired neuromyotonia syndrome. Treatment with estazolam and gabapentin did not improve her symptoms. During hospitalization, her blood sodium level progressively declined (minimum of 114 mmol/L) without reduced food intake, vomiting, and diarrhea. We intravenously administered 3% sodium chloride, which did not improve her condition. Further questioning revealed that the patient had a history of rash in both lower limbs. She had bought a cream 4 months earlier and started applying it to her lower limbs, but discontinued its use 2 months before admission. Further testing showed a 24-hour urine K^+^ level of 37.68 mmol, Na^+^ level of 218.40 mmol, and Cl^-^ level of 215.68 mmol. Toxicological blood analysis by atomic fluorescence spectrometry showed the level of mercury was 0.07 μmol/L (normal level: <0.05 μmol/L). Her urinary mercury level measured using the cold atomic absorption method was 217.50 μmol/mol creatinine, which was considerably higher than the reference range (0–2.25 μmol/mol creatinine for people not in contact with mercury, 0–20 μmol/mol creatinine following long-term exposure). Upon further testing, a high level of mercury (10,572 mg/kg) was detected in the patient's cream. Accordingly, this patient was diagnosed with mercury poisoning, and 2,3-dimercapto-1-propanesulfonic acid (DMPS) treatment was initiated. Her urinary mercury level decreased to 9.67 μmol/mol creatinine, and her neuromyotonia syndrome and hyponatremia were relieved, with urine protein completely disappearing after 3 months of treatment. After excluding other autoimmune diseases and tumors as possible causes, we assumed that mercury poisoning caused acquired neuromyotonia syndrome.

Video 1 (see attachment) shows the clinical manifestations in the nervous system of patients with mercury poisoning.

## 3 Discussion

Acquired neuromyotonia syndrome, also known as Isaacs’ syndrome, is a rare condition of spontaneous and continuous muscle fiber activity of peripheral nerve origin.^[1]^ There are no specific published EMG or clinical diagnostic criteria for this disorder. It is distinguished by clinical manifestations and EMG features. The main clinical manifestations are muscle twitching at rest, cramps, and an undulating wavelike movement visible on the muscle surface.^[8]^ In addition to the classical muscle symptoms, there is a spectrum of autonomic and central nervous system symptoms, including paresthesia, pain, excessive sweating, tachycardia insomnia, personality and mood changes, anxiety, and depression. The EMG shows continuous muscle activity occurring at rest and unaffected by local nerve blockade. A total of 45% to 50% of cases are associated with antibodies that immunoprecipitate voltage-gated potassium channels (VGKCs),^[9]^ mainly including contactin-associated protein 2 (CASPR2) and leucine-rich glioma inactivated 1 (LGI1). It primarily occurs in patients with immune diseases and tumors, and reported cases of mercury poisoning are rare.

We only found 1 case of mercury poisoning with acquired neuromyotonia syndrome. This patient was also from China.^[10]^ He had a history of cooking Tibetan medicine for 6 months, which contains mercury. He developed continuous muscle fiber activity persisting during sleep, accompanied by a burning sensation in his lower limbs 3 months after he stopped contacting Tibetan medicine. He received high-dose dexamethasone therapy and analgesic therapy, but it was ineffective. Further inspection revealed that serum LGI1 and CASPR2 antibodies were positive, blood mercury (2.7 ng/mL) and urine mercury levels (28.7 μmol/mol) exceeded the standard, and the EMG showed myokymic discharges. The patient received chelation treatment and hormone treatment again, and the symptoms were relieved. Owing to the persistent muscle tremor and typical EMG, the patient was diagnosed with acquired neuromyotonia syndrome. After excluding the possibility of immune diseases and tumors, mercury poisoning was considered as the primary cause. Thus, we did not test for LGI1 and CASPR2 antibodies or evaluate the cerebrospinal fluid. Acquired neuromyotonia syndrome was relieved after mercury removal. From our experience, acquired neurosis syndrome caused by mercury poisoning is effectively cured through mercury removal, and hormone therapy may not be beneficial regardless of LGI1 and CASPR2 antibody positivity; however, further clinical observation is needed.

The magnitude of the effects of mercury poisoning can vary considerably depending on the dose and route of exposure. The main cause of poisoning in our patient was the external use of a cream containing mercury, on both lower limbs. Her muscle twitching mainly occurred in the area of mercury exposure, rather than throughout the body. Acquired neuromyotonia syndrome occurred several months after mercury exposure, and it was still present after the exposure had stopped. Our patient was admitted 2 months after discontinuing mercury exposure, while the patient in the other case reported by Aiqing Li was admitted after 3 months.

In addition to neurological symptoms, our patient showed renal lesions. Acute mercury exposure often causes acute renal tubular necrosis and renal failure,^[11–14]^ whereas chronic mercury exposure could induce membranous nephropathy and minimal change nephropathy.^[15–19]^ Our patient presented with obvious hyponatremia caused by renal tubule damage, which was improved by DMPS. Whether mercury poisoning specifically damages the renal tubular sodium channels requires further research.

Although mercury has been used commercially and medically for centuries, it has been banned as a heavy metal in many countries because of its serious negative effects on health. In developing countries, people are still exposed to mercury-containing compounds, particularly in some folk prescriptions^[15,20,21]^ and illegal skin lightening cosmetic products.^[18,22]^ Mercury poisoning caused by these products is insidious and difficult to detect. Nervous system symptoms typically appear weeks or months after mercury exposure and may cause lasting harm to health due to delayed diagnosis. Mercury poisoning is a treatable disease; however, if exposure continues and the patient is not treated, serious complications can occur.^[23]^ Therefore, studies are needed to improve the understanding of the clinical manifestations of mercury poisoning and to develop approaches for detecting mercury exposure in suspected patients.

In summary, mercury poisoning should be considered in patients with neuromyotonia. Early detection of mercury poisoning could prevent unnecessary examination and treatment.

## Acknowledgments

We would like to thank Editage (www.editage.cn) for English language editing.

## Author contributions

**Conceptualization:** Enrong Ran and Yuanjun Liu

**Investigation:** Maohe Wang.

**Supervision:** Yanxia Yi.

**Writing – original draft:** Enrong Ran

**Writing – review & editing:** Enrong Ran, Maohe Wang, Yanxia Yi, Mei Feng, and Yuanjun Liu

## Supplementary Material

Supplemental Digital Content
